# Sequence Analysis and Serological Responses against *Borrelia turicatae* BipA, a Putative Species-Specific Antigen

**DOI:** 10.1371/journal.pntd.0002454

**Published:** 2013-09-19

**Authors:** Job E. Lopez, Hannah K. Wilder, William Boyle, L. Brock Drumheller, Justin A. Thornton, Bridget Willeford, Timothy W. Morgan, Andrea Varela-Stokes

**Affiliations:** 1 Department of Biological Sciences, Mississippi State University, Starkville, Mississippi, United States of America; 2 Department of Laboratory Animal Resources and Care, College of Veterinary Medicine, Mississippi State University, Starkville, Mississippi, United States of America; 3 Department of Pathobiology and Population Medicine, College of Veterinary Medicine, Mississippi State University, Starkville, Mississippi, United States of America; 4 Department of Basic Sciences, College of Veterinary Medicine, Mississippi State University, Starkville, Mississippi, United States of America; Medical College of Wisconsin, United States of America

## Abstract

**Background:**

Relapsing fever spirochetes are global yet neglected pathogens causing recurrent febrile episodes, chills, nausea, vomiting, and pregnancy complications. Given these nonspecific clinical manifestations, improving diagnostic assays for relapsing fever spirochetes will allow for identification of endemic foci and expedite proper treatment. Previously, an antigen designated the *Borrelia* immunogenic protein A (BipA) was identified in the North American species *Borrelia hermsii*. Thus far, BipA appears unique to relapsing fever spirochetes. The antigen remains unidentified outside of these pathogens, while interspecies amino acid identity for BipA in relapsing fever spirochetes is only 24–36%. The current study investigated the immunogenicity of BipA in *Borrelia turicatae*, a species distributed in the southern United States and Latin America.

**Methodology/Principal Findings:**

*bipA* was amplified from six isolates of *Borrelia turicatae*, and sequence analysis demonstrated that the gene is conserved among isolates. A tick transmission system was developed for *B. turicatae* in mice and a canine, two likely vertebrate hosts, which enabled the evaluation of serological responses against recombinant BipA (rBipA). These studies indicated that BipA is antigenic in both animal systems after infection by tick bite, yet serum antibodies failed to bind to *B. hermsii* rBipA at a detectable level. Moreover, mice continued to generate an antibody response against BipA one year after the initial infection, further demonstrating the protein's potential toward identifying endemic foci for *B. turicatae*.

**Conclusions/Significance:**

These initial studies support the hypothesis that BipA is a spirochete antigen unique to a relapsing fever *Borrelia* species, and could be used to improve efforts for identifying *B. turicatae* endemic regions.

## Author Summary

Undiagnosed febrile illnesses continue to afflict those in resource poor countries. Relapsing fever spirochetes are one such pathogen causing a significant health burden, yet the pathogenesis, ecology, and distribution of *B. turicatae* is understudied. To address these shortcomings, we analyzed the amino acid sequence of the *Borrelia* immunogenic protein A (BipA) in isolates of *B. turicatae*. Mice and a canine were also infected by tick bite and transmission and serological responses were evaluated in these two likely mammalian hosts. *B. turicatae* was visualized within the blood of both animals and antibody responses generated against recombinant BipA indicated that the antigen that may be unique to infections caused by *B. turicatae*. Moreover, mice continued to generate antibodies a year after tick bite, suggesting a persistent infection. Our results indicate that the immune responses generated against BipA could identify additional vertebrate hosts, define endemic foci for *B. turicatae*, and increase the awareness of the disease to improve healthcare.

## Introduction

Three causative agents of tick-borne relapsing fever borreliosis in the New World are *Borrelia hermsii*, *Borrelia parkeri*, and *Borrelia turicatae*, with *B. hermsii* being the most epidemiologically and ecologically characterized species [1]. While *B. hermsii* is distributed in high elevation coniferous forests and maintained in enzootic cycles with rodents as the primary reservoir, less is known regarding the other two species. Moreover, few epidemiological studies have been performed and little molecular data exists for *B. turicatae* and its arthropod vectors *Ornithodoros turicata.* There are endemic foci for *B. turicatae* in Texas and Florida, where clinical isolates have been obtained from sick dogs [2], [3], which suggests a role for wild canids in the maintenance of the spirochetes in nature. Dr. Oscar Felsenfeld also reported the distribution of *O. turicata* into Mexico, Central, and South America [4], yet given the absence of Latin American isolates for *B. turicatae* the identification of endemic foci is unclear.

A limitation in defining the distribution of *B. turicatae* has been the absence of diagnostic antigens specific for the species. Previously, the *Borrelia* immunogenic protein A (BipA) of *B. hermsii* was demonstrated to discriminate infections caused by Lyme and relapsing fever borreliosis. Outside of relapsing fever spirochete spp. a homologue of BipA has not been cataloged in the GenBank database [5]. With 36% amino acid identity between *B. hermsii* and *B. turicatae* BipA [5], it is also unclear if the *B. turicatae* homologue induces a host antibody response during infection. This study investigated the sequence similarity of BipA between *B. turicatae* isolates, and we developed a tick transmission system for the spirochetes to determine the antigenicity of recombinant BipA (rBipA) during rodent and canine infections. Collectively, these results suggests that BipA can be used as a diagnostic antigen for *B. turicatae*.

## Methods

### Ethical statement

All animal studies were in accordance with the Mississippi State University Institutional Animal Care and Use Committee (IACUC protocol #'s 11-091 and 12-067). Animal husbandry was provided by veterinary staff and technicians within the Association for Assessment and Accreditation of Laboratory Animal Care and the National Institutes of Health Office of Laboratory Animal Welfare assured program at Mississippi State University. All work was performed in adherence to the United States Public Health Service Policy on Humane Care and Use of Laboratory Animals and the Guide for the Care and Use of Laboratory Animals.

### Amplification and sequence analysis of BipA from *B. turicatae* isolates

*B. turicatae* was cultivated in mBSK medium containing 12% rabbit serum [6], [7]. Amplification and sequencing primers for *bipA* were designed using the 91E135 isolate of *B. turicatae* (Table 1). Additional samples were water (negative control), 95PE-570, 95PE-1807, TCB-1, TCB-2, and FCB-1 isolates [2]. Polymerase chain reaction (PCR) was performed as previously described using the GoTaq Flexi DNA Polymerase (Promega Corporation, Madison, WI, USA). Amplicons were electrophoresed on a 1% agarose gel to visualize the DNA fragment and processed through the QIAquick PCR Purification kit (Qiagen, Germantown, MD, USA), and sequencing performed at Biodesign Institute (Arizona State University, Phoenix, AZ, USA). Nucleotide sequences were analyzed with the Vector NTI software (Life Technologies, Carlsbad, CA, USA), and deposited to GenBank under accession numbers KC845527-KC845531.

**Table 1:** Oligonucleotides and quantitative PCR probe.

Primer	Sequence (5′→3′)	Role
*bipA* F D-Topo	CACCATGTGGTTTGTAAGGAGGGTGGATAT	Amplification and sequencing
*bipA* R Topo	ATTTGTAGCTTCAATTTTAGATTTAAACCCTA	Amplification and sequencing
*bipA F1*	GTTTTCCAAAATTTAGGAGATACTGGTACT	Sequencing
*bipA F2*	TATGCTTTTTGGTACGGGAGAGAA	Sequencing
*bipA R1*	GCCTTTACTTGTAACATCAGAACTCAAGA	Sequencing
*bipA R2*	AATTGAATTTATTGAATTTTCATTTTCTGTT	Sequencing
*flaB* probe	YAK-TGCAGGTGAAGGTGCGCAGGTT-BBQ	Quantitative PCR
*flaB* F	CCAGCATCATTAGCTGGATCAC	Quantitative PCR
*flaB* R	GTTGTGCACCTTCCTGAGC	Quantitative PCR

### Tick colony

The ticks used in the study originated from 12 uninfected adults initially maintained at the Rocky Mountain Laboratories. A cohort of second nymphal stage *O. turicata* was infected by first needle inoculating a group of 3 three Swiss Webster mice with *B. turicatae* 91E135 [2], and uninfected ticks were allowed to engorge on the animals. After molting, vector colonization was confirmed by dissecting the midgut and salivary glands from five ticks and performing immunofluorescent assays (IFA) as previously described [8]. Chicken serum generated by Cocalico Biologicals Inc. against *B. turicatae* recombinant flagellin (rFlaB) was used to detect spirochetes and the secondary antibody was goat anti-chicken IgY Alexa Fluor 568 (Life Technologies, Grand Island, NY, USA).

### Transmission of *B. turicatae*

Prior to the transmission studies, pre-infection serum samples were collected from all animals. Cohorts of five infected ticks per animal were allowed to feed to repletion. The animals used in the study were 10 outbred Swiss Webster mice (Harlan Laboratories Inc., Tampa, FL, USA) and a one-year-old Bluetick hound (Marshall Bioresources, North Rose, NY, USA). For 16 consecutive days, animals were monitored for clinical symptoms and a drop of blood was collected from the mice by tail nick and from the canine's cephalic vein to visualize spirochetes. The day following the first and second febrile episode, 3 ml of blood were collected from the canine for a complete blood count and serum chemistry profile.

Of the mice used in the initial transmission study, four were maintained for one year to assess long-term serological responses generated against BipA. Prior to serum collection for immunoblotting, 2.5 µl of blood was collected for 10 consecutive days and placed into 47.5 µl of SideStep Lysis and Stabilization Buffer (Agilent, Santa Clara, CA, USA). Quantitative PCR (qPCR) was performed as previously described [9] to determine the presence of circulating spirochetes. The probe (*flaB* probe) and primer set (*flaB* F and *flaB* R) used for qPCR were designed for *B. turicatae* flagellin (Table 1).

Two groups of three mice were also needle inoculated intraperitoneally with 1×10^5^ TCB-1 or FCB-1 spirochetes. Infection was confirmed by dark field microscopy and serum samples were collected one month after inoculation to determine reactivity to rBipA expressed from the 91E135 isolate.

### Expression of *B. turicatae bipA* as a recombinant fusion protein, serological and linear regression analysis

To express *bipA* from *B. turicatae* 91E135 as a 75 kDa thioredoxin and histidine tagged fusion protein in *Escherichia coli*, the gene was amplified as previously described [5] using *bipA* F Topo and *bipA* R Topo primers (Table 1). The amplicon was cloned into the pET 102/Directional TOPO expression vector following the manufacturer's instructions (Life Technologies). Top10 *E. coli* were transformed, plasmid DNA isolated, and sequence analysis using *bipA F1*, *bipA F2*, *bipA R1*, *bipA R2* primers (Table 1) was performed as previously described [5] to determine if an error had been introduced during amplification. To produce recombinant protein, BL21 *E. coli* were transformed with the *bipA* expression vector following the manufacturer's instructions, and induction was performed with 1 mM IPTG. rBipA was purified using the Ni-MAC Purification system (Novagen, Durmstadt, Germany).

Immunoblotting was performed to evaluate the immunogenicity of rBipA during *B. turicatae* infections. Protein lysates from 1×10^8^ spirochetes, 1 µg of *B. hermsii* rBipA , and 1 µg *B. turicatae* rBipA were electrophoresed and transferred to polyvinylidene fluoride (PVDF) membranes using TGX gels, the Mini-PROTEAN Tera cell, and the Mini Trans Blot system (BioRad, Hercules, CA, USA). Pre- and post-infection serum samples were evaluated by immunoblotting at a 1∶500 dilution and the secondary molecule used was Rec-protein G-HRP (Life Technologies) at a 1∶4,000 dilution. Immunoblots were also probed with the Anti-polyHistidine Peroxidase monoclonal antibody (Sigma-Aldrich, St. Louis, MO, USA) at a 1∶4,000 dilution. Titers against rBipA were determined by immunoblotting with serum dilutions ranging from 1∶500 to 1∶125,000.

Linear regression analysis was performed by calculating the density of rBipA protein bands from immunoblots probed with serum samples diluted from 1∶800 to 1∶12,800. Serum samples included Anti-polyHistidine Peroxidase monoclonal antibody (Sigma-Aldrich, St. Louis, MO, USA) and serum samples from mice and the canine infected by tick bite. ImageJ, http://imagej.nih.gov/ij (National Institutes of Health, Bethesda, Maryland, USA), was used to analyze digitally scanned immunoblots and the density of each protein band was calculated. The R software package, www.r-project.org, was used to calculate equations of regression, R^2^ values, and significance.

### Histopathology

One year after tick bite, mice were euthanized by isoflurane inhalation followed by cervical dislocation and tissues were placed in 10% neutral buffered formalin for fixation. Sections of brain and synovial joints from the front and rear leg were processed and embedded in paraffin using standard histologic techniques. Paraffin embedded tissues were cut in 5 µm sections, deparaffinized, adhered to glass slides, and stained with hematoxylin and eosin (H&E), or silver stained using standard histologic techniques. The sections were examined by light microscopy for inflammatory and degenerative lesions in the cerebrum, cerebellum, brainstem, and synovial joints by a board certified veterinary pathologist at Mississippi State University.

## Results and Discussion

**Figure 1:**
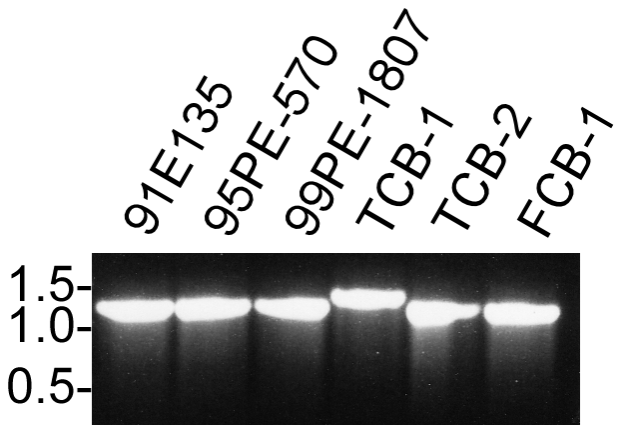
PCR analysis of *bipA* from six *B. turicatae* isolates. Molecular masses are shown in kilobases to the left of the gel.

**Figure 2:**
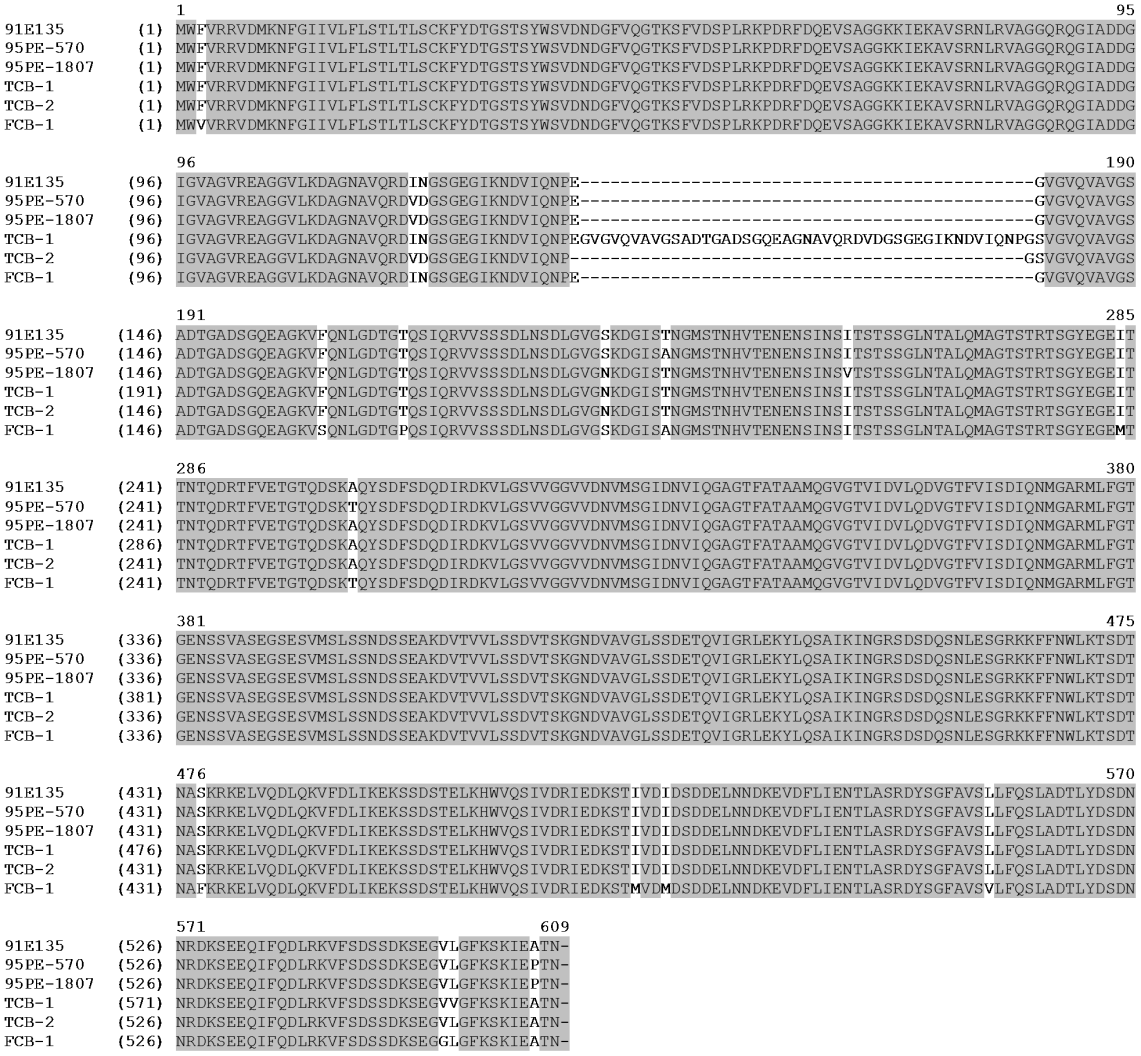
Amino acid alignments of *B. turicatae* BipA from six isolates. Identical amino acids are shaded grey.

BipA was originally identified by an immunoproteomic antigen discovery approach [5], [10]. The degree dissimilarity between *B. hermsii* and *B. turicatae* homologues prompted our investigation to compare sequences between *B. turicatae* isolates, and to evaluate the protein's immunogenicity in two mammals likely to be naturally exposed to the spirochetes. PCR amplification of *bipA* from three canine (FCB-1, TCB-1, and TCB-2) and three tick (91E135, 95PE-570, and 95PE-1807) isolates produced a product of the expected molecular mass, with TCB-1 producing a slightly larger amplicon (Figure 1). Sequence analysis identified an additional 135 nucleotides encoding 45 amino acids for TCB-1 (Figure 2). While there was an overall 89% amino acid identity of BipA between isolates, the sequences flanking the 45 additional amino acids of TCB-1 were 97% identical. A similar observation was reported for *B. hermsii* BipA [5]. The protein from genomic group II (GG II) isolates of *B. hermsii* contained five regions of 3–24 amino acid insertions when compared to genomic group I (GG I) isolates. Furthermore, the amino and carboxy terminus of BipA between GGI and GGII isolates shared the highest degree of conservation.

**Figure 3:**
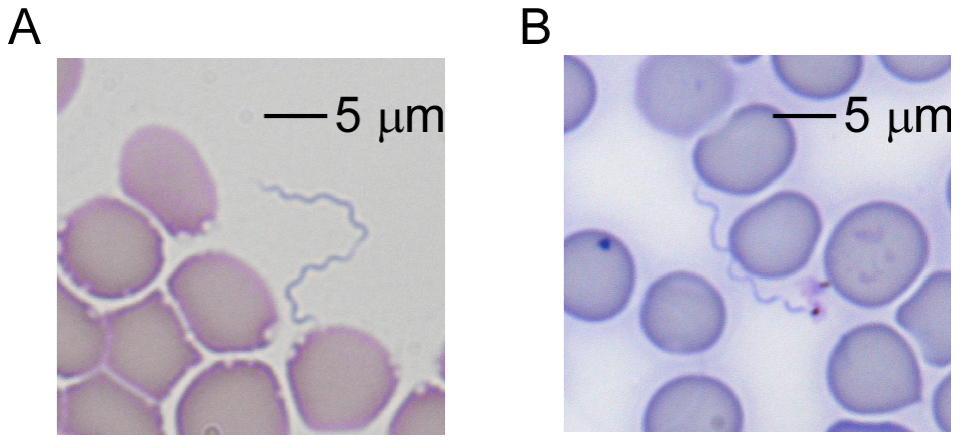
Visualization of *B. turicatae* within canine (A) and murine (B) blood after staining thin smears by Wright-Giemsa. A 5 µm scale is shown.

Currently, the only known isolates of *B. turicatae* originate from argasid soft ticks and sick dogs [2]. Furthermore, the mammalian hosts for most species of relapsing fever spirochetes include rodents and insectivores [11]. This knowledge directed us to evaluate the antigenicity of *B. turicatae* rBipA after tick bite using canine and murine animal models. To establish an infected tick colony, uninfected *O. turicata* engorged on a Swiss Webster mouse needle-inoculated with *B. turicatae* 91E135. Spirochete colonization was confirmed by performing IFA on the midgut and salivary glands after the ticks molted (data not shown). The remaining infected ticks fed to repletion on Swiss Webster mice and a Bluetick hound, and within four and eight days after tick bite spirochetes were visualized in murine and canine blood, respectively (Figure 3 A and B). While the mice remained active when *B. turicatae* were visualized in the blood, the canine became febrile, lethargic, and following a given spirochetemic episode, acutely thrombocytopenic (Table 2). *B. turicatae* repopulated the blood from both groups of animals within four days after the initial spirochetemia, after which bacteria were undetectable by microscopy.

**Table 2:** Canine temperature and platelet counts during infection.

Days post infection	Temperature (Celsius)	Platelet Counts per µl^A^
0	39.0^B^	331,000^B^
5	39.0	-
6	39.0	-
7	39.1	-
8^C^	40.4	-
9	38.4	6,000
10	38.9	-
11	38.6	-
12^C^	40.1	-
13	38.8	8,000
14	38.4	-
15	38.3	-
16	38.1	204,000

A Values are based on automated platelet counts. Reference range was from 160,000 to 650,000. Automated platelet counts were noted on days 9 and 13, when counts were below normal.

B Baseline temperature and platelet counts were taken prior to transmission by tick bite.

C Spirochetemic episode.

**Figure 4:**
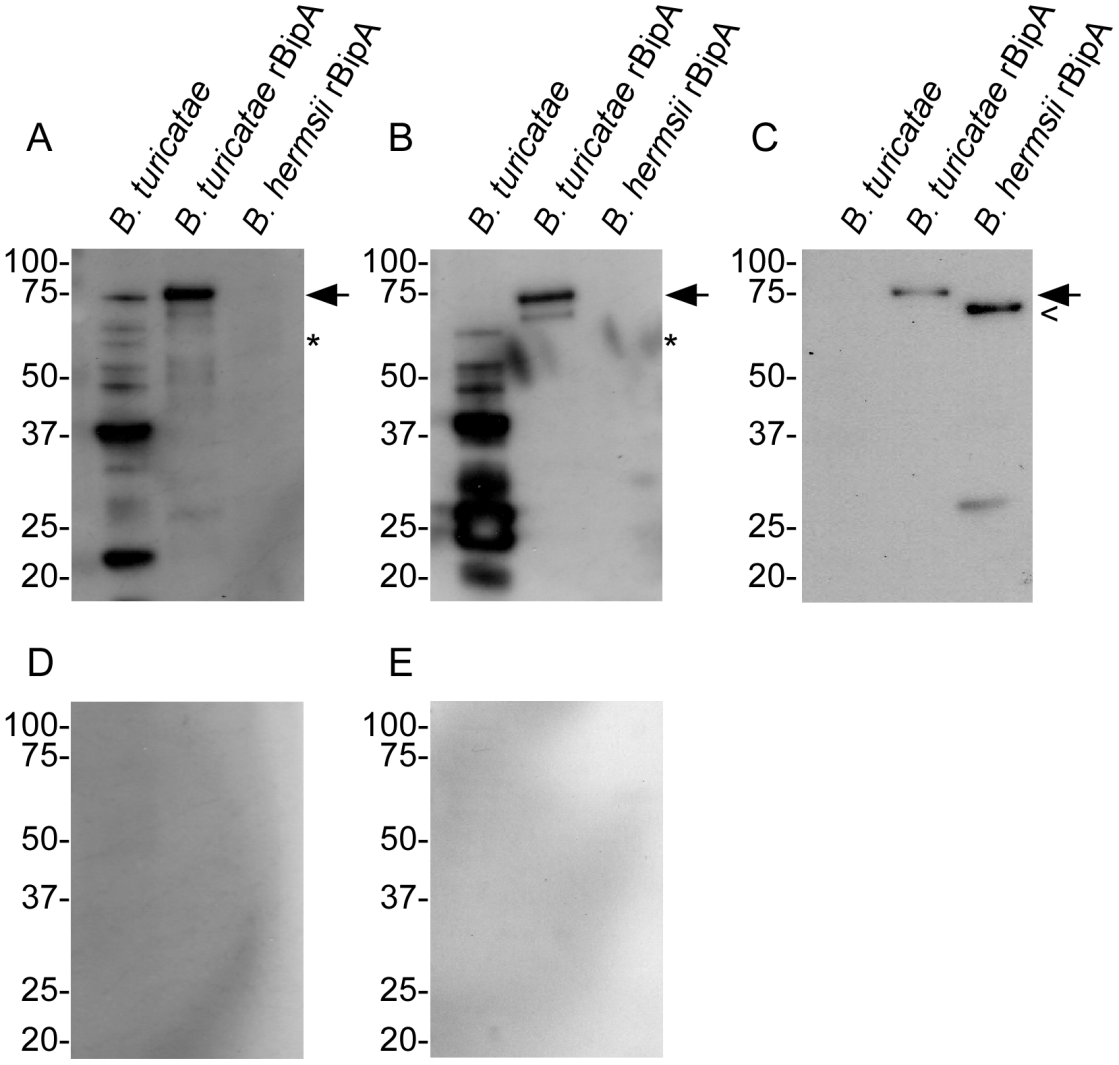
Immunoblotting to evaluate serological responses from an infected canine (A) and mice (B) against *B. turicatae* protein lysates and rBipA. Immunoblots were also probed with an anti-polyhistidine monoclonal antibody (C), canine pre-infection serum (D), and murine pre-infections serum (E). The (←), (*), and (<) indicate the molecular size of *B. turicatae* rBipA, native *B. turicatae* BipA, and *B. hermsii* rBipA, respectively. Molecular masses are indicated to the left of each immunoblot in kilodaltons.

**Figure 5:**
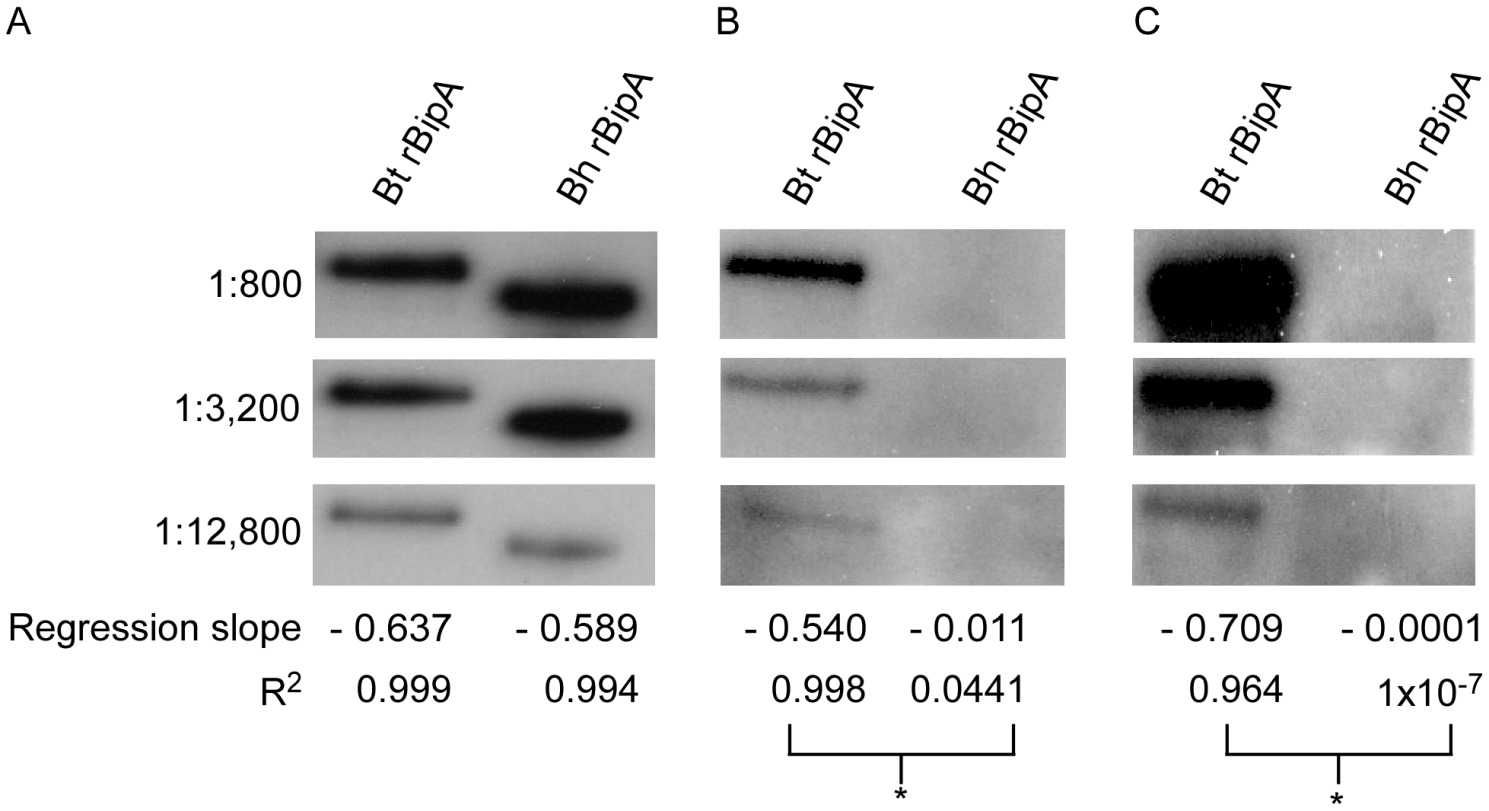
Linear regression analysis of serological responses generated against *B. turicatae* rBipA. Immunoblots containing *B. turicatae* rBipA (Bt rBipA) and *B. hermsii* rBipA (Bh rBipA) were probed with an anti-polyhistidine monoclonal antibody (A), and with serum from two mice infected by tick bite (B and C). Serum dilutions, regression slopes, and R^2^ values are show on the far left and below each immunoblot, respectively. The (_*_) indicates significant differences between regression slopes and R^2^ values.

Producing rBipA using the same expression vector as *B. hermsii bipA* [5] indicated that infected animals generated an immunological response that recognized *B. turicatae* rBipA, yet antibody binding against the recombinant *B. hermsii* homologue was undetectable (Figure 4 A and B). Probing the immunoblots with an anti-polyhistidine monoclonal antibody confirmed that similar protein loads of *B. turicatae* and *B. hermsii* rBipA were electrophoresed, while pre-infection serum samples failed to produce a detectable antibody response against *B. turicatae* protein lysates or rBipA (Figure 4 C–E). Canine and murine IgG titers using serum samples collected 8 weeks after tick bite ranged from 1∶12,800 to1∶28,800. Also, regression analysis indicated significant differences (P≤0.05) in slopes and correlation coefficients (R^2^) when immune serum samples were probed against *B. turicatae* and *B. hermsii* rBipA (Figure 5 A–C). These results indicate different affinity characteristics against rBipA from a given species when animals were infected with *B. turicatae*.

**Figure 6:**
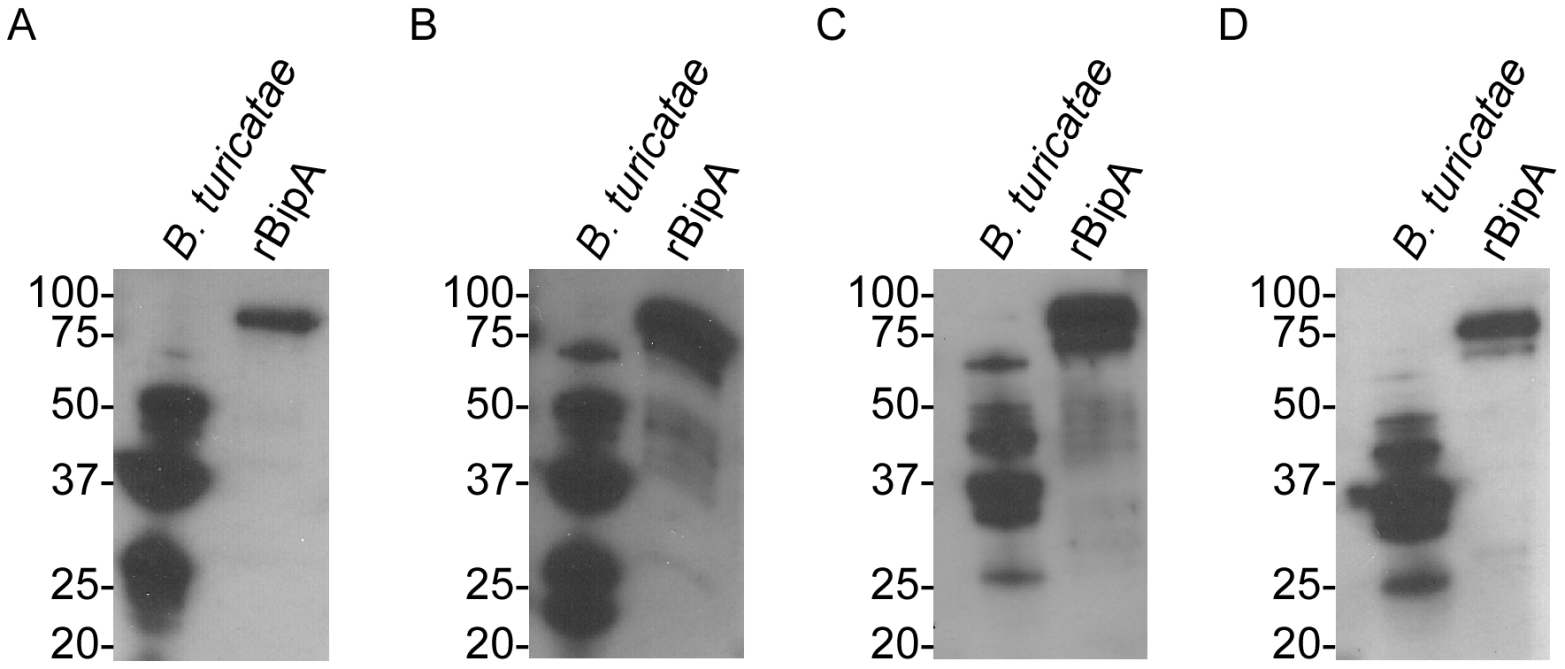
Serological responses from mice infected with TCB-1 (A and B) and FCB-1 (C and D). *B. turicatae* protein lysates and rBipA produced from the 91E135 isolate are shown. Molecular masses are indicated to the left of each immunoblot in kilodaltons.

With BipA from TCB-1 and FCB-1 being the most divergent to the 91E135 homologue, we evaluated serological responses against rBipA from animals infected with TCB-1 and FCB-1. Inoculating mice with each isolate determined that antibodies generated against TCB-1 and FCB-1 BipA were cross reactive against rBipA from *B. turicatae* 91E135 (Figure 6 A–D). Similarly, in *B. hermsii* there was sufficient amino acid conservation between BipA from *B. hermsii* GGI and GGII isolates that mice infected with GG II isolates produced a detectable serological response to rBipA that was expressed from a GG I isolate [5]. Collectively, these results suggest that BipA may be a unique antigen for the given species of relapsing fever spirochete causing infection.

**Figure 7:**
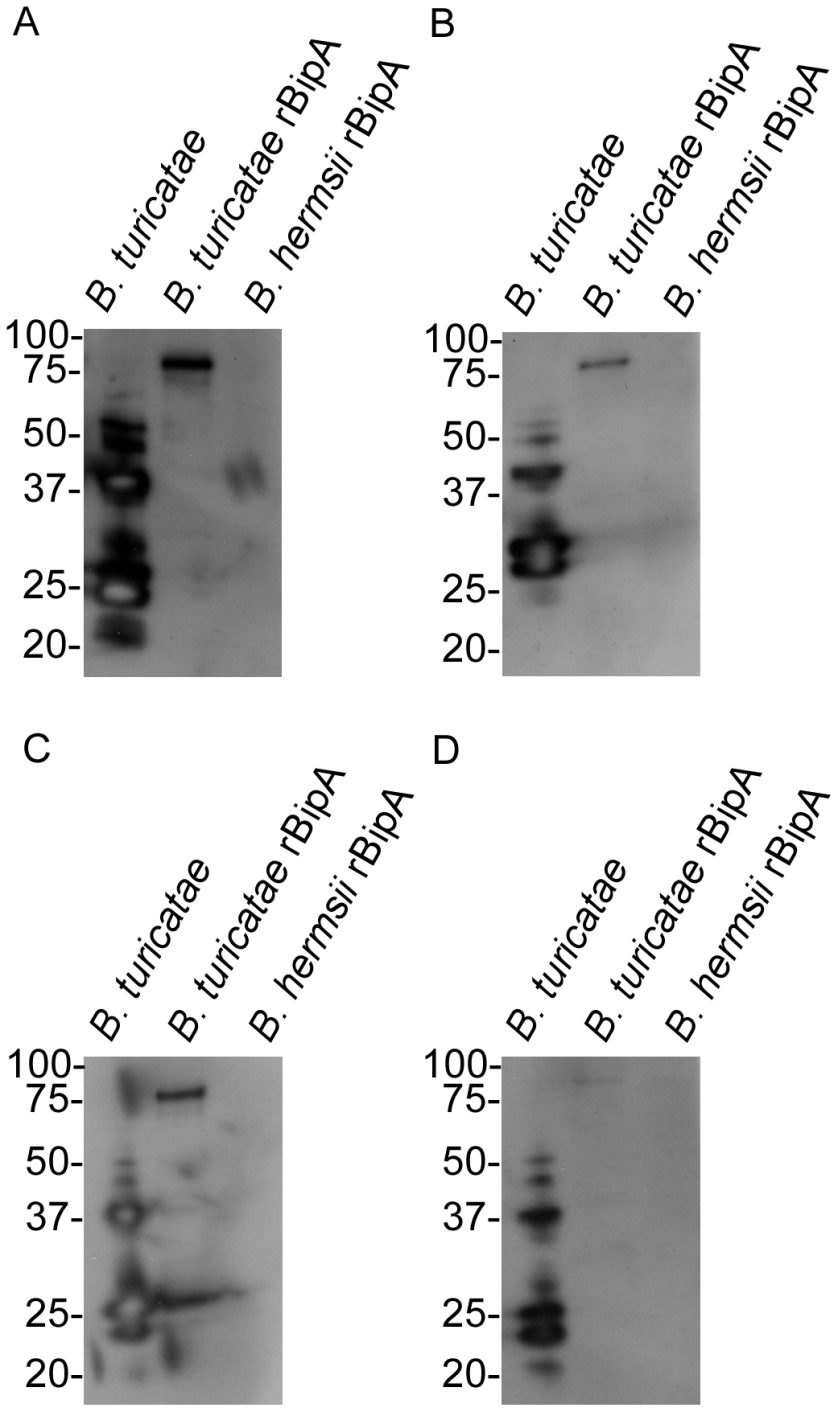
Long-term serological responses from four mice one year after tick bite (A–D). *B. turicatae* protein lysates and rBipA produced from the 91E135 isolate and *B. hermsii* are shown. Molecular masses are indicated to the left of each immunoblot in kilodaltons.

Previous studies by Cadavid *et al*. reported that BALB/c and SCID mice needle inoculated with the Ozona isolate of *B. turicatae* developed long-term infections of the brain and joints [12]. Given the persistent nature of the spirochetes within rodents, IgG responses in mice were evaluated one year after transmission by tick bite. Prior to serological analyses, qPCR performed on murine blood samples collected for 10 consecutive days indicated that the mice were no longer spirochetemic (data not shown). Immunoblotting demonstrated that three of four mice continued to generate an IgG response against rBipA one year after the initial exposure, while one animal produced a weakly detectable response (Figure 7 A–D). Interestingly, *B. turicatae* is no longer detected within the blood of Swiss Webster mice after approximately 14 days after tick bite (data not shown), and with a serum half-life of 20–30 days for IgG, these results suggest a persistent infection and antigen exposure to the host immune response.

Central nervous system (CNS) infections by relapsing fever spirochetes vary between species and genetic variants. *Borrelia duttonii* can reemerge in the blood from the brain after a period of quiescence [13]. Serotype A of *B. turicatae* Ozona were neurotropic in mice, while animals infected with serotype B spirochetes colonize the joints and heart [12], [14], [15]. Interestingly, CNS infection caused by *B. duttonii* and serotype A of *B. turicatae* Ozona failed to produce noticeable tissue damage [12], [13]. In our study, postmortem necropsies of mice one year after infection by tick bite did not identify inflammatory or degenerative changes within the cerebrum, cerebellum, brain stem, or diarthrodial joints of the fore or hind limbs (data not shown). Spirochetes were also undetectable in tissue sections, and it was unclear if the animals were still infected at the time of euthanasia. However, the persistent antibody responses generated against rBipA can be targeted to increase the likelihood of determining if an animal has been exposed to the spirochetes.

With results suggesting that BipA may be a species-specific antigen, additional studies should evaluate homologues from less characterized yet closely related species to *B. turicatae*. For example multilocus sequencing indicated that *Borrelia johnsonii*, a novel species of relapsing fever spirochete that colonizes *Carios kelleyi* [16], was closely related to *B. turicatae* and *Borrelia parkeri.* As additional sequence information is obtained from *B. johnsonii* and *B. parkeri* and animal models developed, the diagnostic potential of BipA can be further evaluated as an antigen unique to a given species of relapsing fever spirochete.

The maintenance and ecology of *B. turicatae* in the southern United States and Latin America is poorly understood, and given the nonspecific clinical symptoms, the disease is likely under reported. Historically, mapping endemic foci has been associated with capturing ticks at sites where human infection occurred and evaluating the arthropods for spirochete colonization, or by obtaining clinical isolates from sick dogs [2], [3], [17]. Pathogen surveillance based on identifying infected ticks can be difficult because *O. turicata* are nest-, den-, and cave-dwelling with a 5–60 minute bloodmeal [3], [11], [18], and the ticks are rarely identified on the host. We are also unaware of serological surveys for *B. turicatae* probably due to the degree of antibody cross-reactivity that occurs during spirochete infections [5], [19], [20]. Given the characterization of BipA, serological analyses to identify endemic foci for *B. turicatae* where rodents and wild canids are monitored as sentinels are possible.
